# Health-related quality of life, psychological issues and concerns among sarcoma survivors: a mixed method study

**DOI:** 10.3332/ecancer.2024.1657

**Published:** 2024-01-22

**Authors:** Arti Suhag, Kamlesh Kumari Sharma, Surya Kant Tiwari, Poonam Joshi, Sameer Rastogi, Simran Kaur

**Affiliations:** 1 College of Nursing, All India Institute of Medical Sciences, New Delhi 110608, India; 2 College of Nursing, All India Institute of Medical Sciences, Bathinda, Punjab 151001, India; 3 College of Nursing, All India Institute of Medical Sciences, Raebareli, Uttar Pradesh 229405, India; 4 College of Nursing, All India Institute of Medical Sciences, Kalyani, West Bengal 741250, India; 5 Department of Medical Oncology, All India Institute of Medical Sciences, New Delhi 110608, India; 6 Department of Medical Physiology, All India Institute of Medical Sciences, New Delhi 110608, India; a https://orcid.org/0000-0003-4718-0398; b https://orcid.org/0000-0002-7016-8437

**Keywords:** anxiety, body image, depression, fertility, marriage, nursing care, personal satisfaction, quality of life, sarcoma, stress, survivorship

## Abstract

**Purpose:**

We aimed to explore the health-related quality of life (HRQoL), psychological issues and concerns among sarcoma survivors in India and assess their satisfaction with nursing care.

**Methods:**

This study employed a sequential mixed-methods design, enrolling 100 sarcoma survivors from July to December 2021, with data collected using standardised questionnaires for HRQoL, depression, anxiety, stress, cognitive impairment and self-structured satisfaction with nursing care. Qualitative data were gathered through focused group discussions.

**Results:**

The mean global health score among sarcoma survivors was 79.48 ± 16.26. A significant number of survivors had symptoms of mild-to-moderate depression (30%), severe anxiety (12%), stress (16%) and mild cognitive impairment (5%). Significant mean rank differences were observed between anxiety and financial difficulty (*p* < 0.05), emotional functioning (*p* < 0.001), cognitive functioning (*p* < 0.001), pain (<0.05), insomnia (*p* < 0.001), fatigue (*p* < 0.001), anorexia (*p* < 0.05) and nausea/vomiting (*p* < 0.001). Educational qualification had a significant association with depression and anxiety while family history of cancer emerged as a significant factor associated with anxiety and stress among survivors. Qualitative analysis revealed themes related to body image, societal discrimination, socio-economic impact, marriage concerns and fertility issues. Survivor satisfaction with nursing care was good.

**Conclusion:**

A substantial number of sarcoma survivors had an average HRQoL and experienced depression, anxiety and stress. Our study emphasizes the importance of holistic survivorship care, involving nurses in post-treatment support, and addressing societal discrimination and psychosocial concerns to enhance their quality of life.

**Implications for cancer survivors:**

Our study calls for a holistic approach to sarcoma survivor care and emphasizes the importance of personalised survivorship care plans led by nurses to address the diverse needs of sarcoma survivors in India. Such plans should encompass strategies for managing depression, anxiety and stress, along with addressing body image concerns and social support.

## Introduction

A sarcoma is a rare kind of cancer that can affect individuals of all age groups, including adolescents and young adults. Sarcomas are distinct from other carcinomas, as they develop in the connective tissue cells that support various tissues in the body. There are more than 50 types of sarcomas, which are broadly categorised as soft tissue sarcomas and bone sarcomas. In India, osteosarcoma is the most common bone sarcoma, followed by Ewing sarcoma and Chondrosarcoma [1]. The commonly involved sites are bones, muscles, tendons, cartilage, nerves, fat and blood vessels of arms and legs, but can also occur in other areas of the body [2].

Various studies have reported that the health-related quality of life (HRQoL) of sarcoma patients was significantly limited, particularly in the functioning scales [3, 4]. A qualitative study found that survivors faced challenges related to altered body image, mobility limitations, poor health and concerns about their lifestyle but were hesitant to discuss these issues with their oncologists [5].

Sarcoma survivors face a variety of challenges, including depression, anxiety, post-traumatic stress disorder and fear of recurrence. A recent meta-analysis showed that mental health had a negative effect on the quality of life (QOL) among chronic cancer survivors [6]. They also face difficulties in returning to work, experience decreased neuro-cognitive abilities and physical functioning; and encounter various issues with verbal reasoning, emotional functioning, pain, fatigue and even thoughts of suicide. Furthermore, survivors' employment, marriage and fertility can be impacted too [7].

We have limited understanding regarding how sarcoma survivors experience continuity of care during their transition from completion of cancer treatment towards recovery, and when and how to best address their needs. In our setup, nurses are involved in the care of sarcoma patients during their hospitalisation.

However, after discharge, when sarcoma survivors attend the specialty clinic, nurses have little to no interaction with them. Furthermore, there is a pressing need to involve nurses in post-treatment cancer care to address their physical and mental needs [8]. There is also a significant underrepresentation of integrative quantitative and qualitative studies exploring sarcoma survivors' concerns, psychological problems, satisfaction with nursing care, and expectations of survivorship care, particularly in the Indian context.

Therefore, we conducted a sequential mixed-method study aimed at exploring the HRQoL, psychological issues and concerns among sarcoma survivors; and assess their satisfaction and expectations with survivorship care.

## Material and methods

### Study design, setting and participants

The present study was based on a sequential mixed-method design conducted among sarcoma survivors at a selected tertiary care facility in Northern India from July to December 2021. It employed a two-phase sequential mixed-methods study: a quantitative study followed by a qualitative study. A total of 100 participants were enrolled using convenient sampling for quantitative study and purposive selection for focused group discussions. The study included participants aged between 15 and 60 years who were attending follow-up in the sarcoma clinic, give written informed consent (>18 years) or assent (15–18 years), and were able to understand and write in Hindi or English.

### Ethical consideration

The study was conducted after getting approval from the Institutional Ethics Committee (vide IECPG-256/24.06.2021) and adhering to the Helsinki Declaration of 1975, as revised in 2013. A detailed explanation regarding the purpose of the study was provided to the study participants before obtaining their written informed consent or assent. Participation in the study was entirely voluntary, and participants were also assured of the confidentiality and anonymity of the obtained information.

### Sample size

The sample size was calculated using the formula, *n* = *Z*² *P* (1−P)/*d*². At a 95% confidence interval, anticipated psychological issues among sarcoma survivors of 50%, and a precision of 10%, the calculated sample size was 100. Data saturation technique was used for qualitative arm of the study for which ten participants were interviewed.

### Tools for data collection

We used a total of five tools consisting of the sociodemographic tool and clinical datasheet, European Organisation for Research and Treatment of Cancer Quality of Life (EORTC QLQ-C30), Depression, Anxiety, and Stress Scale (DASS)-21, mini-mental status examination (MMSE), and a self-developed Satisfaction Related to Nursing Care Scale for quantitative data collection. Permission to use standardised tools was obtained from the respective copyright authors.

#### Tool I: Socio-demographic and clinical datasheet

Tool I comprised a socio-demographic and clinical datasheet, with socio-demographic variables such as age, gender, residence, type of family, education and occupation. The clinical profile of the patients included diagnosis, history of relapse, chemotherapy, radiation therapy and surgery.

#### Tool II: EORTC QLQ-C30

Tool II consisted of the EORTC QLQ-C30 [9] an internationally validated cancer-specific HRQoL instrument. This instrument measures global QOL as well as five functioning (physical, emotional, social, cognitive and role) and nine symptom sub-scales (fatigue, pain, nausea/vomiting, dyspnoea, sleep disturbances, appetite loss, constipation, diarrhoea and financial impact). All scale scores are transformed to a linear scale ranging from 0 to 100.

#### Tool III: DASS-21

Tool III comprised the DASS-21 [10] which is a shorter version of the original 42-item DASS, containing 21 items with adequate psychometric properties and validity as an approved instrument for measuring three self-reported scales: depression, anxiety and stress in adult patients. Each scale consists of seven items measured on a four-point Likert scale from 0 to 3 (0: ‘Did not apply to me at all’, and 3: ‘Applied to me very much or most of the time’). Depression, anxiety, and stress scores are measured by summing the scores of the related items and classified as: ‘normal, mild, moderate, severe, or extremely severe’.

#### Tool IV: MMSE

Tool IV consisted of a MMSE, [11] a 30-point test designed to measure cognitive impairment among sarcoma survivors and classified as normal cognition (> 24), mild cognitive impairment [19–23], moderate impairment [10–18], and severe cognitive impairment (<9).

#### Tool V: Satisfaction Related to Nursing Care Scale

Tool V comprised a self-developed scale for assessing satisfaction related to nursing care, which was validated from experts for all age group population. This scale consisted of ten items that measured the satisfaction of sarcoma survivors with the survivorship care provided by a specialised nurse. Participants rated their satisfaction on a five-point Likert scale, with scores ranging from ‘strongly agree’ [5] to ‘strongly disagree’ [1]. In the present study, Cronbach alpha of the satisfaction with nursing care was 0.759.

### Qualitative sample

A semi-structured focused group interview, pre-tested through a pilot group discussion, was conducted among ten participants, including seven adults and three paediatric participants. Six broad open-ended questions were asked to the participants, and after further triggering, the responses of the survivors were recorded (Supplementary Table 1). To overcome language barriers during the focused group interview, questions were posed in a language understood by the participants.

### Data collection process

The participants were provided with an information sheet describing the aim of the study. After obtaining consent or assent from the survivors, the questionnaire was given either in the form of Google Forms or handouts to the survivors attending the outdoor patient department (OPD). It took almost 30–35 minutes for sarcoma survivors to complete the questionnaires. Subjects were followed up by a specially prepared nurse in survivorship care in person and telephonically for a minimum period of 2 months, from the day of enrolment to the completion of the study. She inquired about the well-being of the survivors and guided them to the respective OPDs based on their complaints. A focused group discussion was conducted with a purposively selected group of 10 out of a total of 100 sarcoma survivors by two members of the research team, keeping the specialised nurse outside to yield unbiased results.

### Analyses

#### Quantitative analysis

The collected data were coded and summarised using Microsoft Excel. Both descriptive and inferential statistics were performed using IBM SPSS Statistics for Windows, version 26.0 (IBM Corp., Armonk, N.Y., USA). Descriptive statistics included frequency, percentage, range, mean and SD. The normal distribution of the variables was evaluated using the Kolmogorov–Smirnov test. Due to the non-normal distribution, a non-parametric test (Mann–Whitney *U*) was used to compare the mean rank difference between EORTC QOL sub-scales and depression, anxiety and stress. Multiple linear regression analysis was conducted to identify factors influencing depression, anxiety and stress among sarcoma survivors. The significance level was set at a *p*-value of ˂0.05.

#### Qualitative analysis

The data were analysed using thematic analysis to explore the concerns of sarcoma survivors and their satisfaction with survivorship care. This method involves analysing the meaning of individual experience through the identification of subtheme and theme. The data consisted of narratives and the verbatim dialogue between the interviewer and interviewee. While coding and deriving themes, participants' responses were translated into English. The final interpretative refinements to the analysed data were made after validation by experts.

## Results

A total of 100 sarcoma survivors participated in the present study. The mean age of the participants was 28.76 ± 12.11 years, with the majority belonging to the age group of 15–30 years (60%). Most of the participants were males (66%) and unmarried (56%). The majority of the sarcoma survivors had either osteosarcoma or Ewing's sarcoma (65%), received chemotherapy (87%), radiation therapy (45%) and undergone surgery (96%) as a treatment modality. A small number of survivors had a family history of cancer (5%) and relapse history of sarcoma (9%) (Table 1). The mean global health/QOL scores among sarcoma survivors were 79.48 ± 16.26, indicating average QOL. In the functioning sub-scales, the needs reportedly low-scored by sarcoma survivors were in the emotional domain, followed by role functioning. Regarding symptoms sub-scale, participants reported experiencing fatigue, pain and financial difficulties (Table 2). A significant number of survivors had symptoms of mild-to-moderate depression (30%), while 5% of the survivors had symptoms of severe depression. Additionally, symptoms of mild-to-moderate anxiety were observed in 16% of the survivors, while 12% exhibited severe anxiety levels. Varying levels of stress were reported by 16% of the survivors, requiring referral and pharmacotherapy. The mean cognitive scores score of sarcoma survivors was 27.54 ± 2.43 with 5% of the survivors exhibiting mild cognitive impairment. The satisfaction of sarcoma survivors with the survivorship care provided by a specialised nurse in the sarcoma clinic is shown in Table 3.

Table 4 depicts the mean rank for DASS-21 components based on EORTC QoL sub-scales. Significant differences were observed between depression and emotional functioning. Furthermore, a significant mean rank difference was evident among financial difficulty, emotional functioning, cognitive functioning, pain, insomnia, fatigue, nausea/vomiting anxiety; and stress. Additionally, a significant mean difference was observed between anorexia and anxiety (Table 4). The results of multiple linear regression analysis examined the sociodemographic factors associated with depression, anxiety and stress among sarcoma survivors. It revealed that residence and educational qualification had a significant association with depression in sarcoma survivors. Additionally, a family history of cancer emerged as a significant factor associated with anxiety and stress among survivors (Table 5).

### Qualitative findings

Six themes were derived from a focused group discussion with sarcoma survivors.

**1) Body image, physical appearance, low self-esteem and acceptance**

Sarcoma survivors often deal with disrupted body image and physical appearance. After surgery, many experience altered body gait, limping in the operated leg, difficulty in performing daily activities, and visible scars on their bodies. These changes can lead to lower self-esteem and feelings of embarrassment due to the alterations in their physical appearance. Several participants responded:

*‘The scar is present on my operated leg, it also looks so slim as compared to my other leg. It goes backward side when I walk which is easily noticed by another person. I feel very bad because my body posture is not normal now.’*

*‘My complete leg was amputated, it looks very awkward. Due to this I feel embarrassed and stressed*

*sometimes because I can’t do my daily activities normally.’*

Some of the survivors had accepted and adjusted to these changes in their body appearance and structure.

*‘My leg is slim, have surgery marks and limping is present in it when I walk. but I don’t bother about it*

*because it’s the reality of my life which I have accepted.’*

**2) Discrimination by society and support from family, relatives and colleagues**

Sarcoma survivors have faced discrimination from society, including hurtful comments and stigmatisation due to cancer diagnosis. Few respondents describe such an instance:

*‘A lot of people did sideline, pass comments, mentally disturbed me, they used to say that this is the sin of his past deeds.’*

*‘Society people used to do discrimination, people talk back and forth that she is having cancer no one willmarry with her.’*

This might have led to a significant psychological burden, aligning with the quantitative findings showing a significant number of survivors experiencing depression, anxiety and stress. Almost all participants reported receiving support from their family, relatives and colleagues.

*‘No one passes wrong comment on me, my family members and relatives supported me with regard to cancer.’*

*‘I didn’t tell to my family, relatives and the society. I am in army I get one attendant from army side. All army people are very supportive they help in a lot.’*

**3) Socio-economic impact of sarcoma**

Sarcoma had caused unemployment, job difficulty, anticipated job loss, discontinuation from education, and psychological stress to the survivors, but few survivors expressed hope, resilience, and determination to continue.

*‘Due to illness I am not getting job anywhere due to which I have to do the farming. I am very stressed due to unemployment.’*

*‘Due to disease my education disturbed, till now I have not thought about job.’*

*‘Difficulty in job will come due to cancer if I will tell them they might not give me job, but I do not give up.’*

**4) Marriage concerns, denial of marriage and fertility issues**

Sarcoma survivors have expressed concerns about marriage, including denial, lack of planning, and uncertainty about the future. Additionally, some of them faced fertility issues due to sarcoma.

*‘I don’t think about marriage because no one will be happy to give me his girl.’*

*‘I do not have children, we are trying to conceive but not able to conceive and treatment is also going for that.’*

**5) Quality of services, amenities and waiting for an hour**

The majority of participants appreciated the quality of service and had an overall positive experience during their treatment at the institute. Some of the participants mentioned long waiting hours for chemotherapy and radiation therapy and faced initial difficulties.

*‘The experience in AIIMS was good but in starting we have to face lot of difficulty, we have to roam around here and there it takes lots of time due to which treatment is delayed.’*

*‘The overall experience is good but the patient who are serious have to wait for long time outside the OPD and they don’t know till what time their turn will come.’*

**6) Satisfaction with the doctor, nurses and healthcare professionals**

Sarcoma survivors expressed satisfaction with the behaviour of the doctors, staff nurses and health care professionals.

*‘The doctor, staff nurse and health care workers help me and supported me during my treatment. They give positive reinforcement to patient’s.’*

*‘The doctor and staff have lot of workload but still they behave very nicely with the patient and help them.’*

## Discussion

The present study was an attempt to explore the HRQoL, psychological burden, concerns and satisfaction with survivorship care among sarcoma survivors in India as well as to redefine the role of the nurse in survivorship care. The major findings of our study revealed that almost half of the participants had an average HRQoL. About one-third of sarcoma survivors experienced some form of depression and anxiety, ranging from mild-to-severe levels. The majority of sarcoma survivors exhibited no cognitive impairment.

The results revealed that sarcoma survivors who were depressed exhibited poor emotional functioning. In addition, survivors who experienced anxiety or stress reported financial difficulties, poor emotional functioning, poor cognitive functioning, increased pain, insomnia, fatigue, and nausea/vomiting compared to their counterparts. Also, anxious sarcoma survivors reported experiencing anorexia. Concerns of sarcoma survivors were in the domains of body image, physical appearance and low self-esteem, acceptance, social support from family, relatives and colleagues, discrimination, socio-economic impact of sarcoma, marriage concern, denial of marriage, and fertility issues. Another important point to be noted in our study is that the majority of survivors belonged to the reproductive age group.

Understanding HRQoL is essential when tailoring treatment options and addressing a patient’s needs. In the present study, we used the EORTC QLQ-C30 to assess HRQoL among sarcoma survivors. Higher mean scores indicate a better QOL for the functioning scales and a higher symptom burden for the symptom scales. When compared with previous studies, [3, 12] sarcoma survivors in our study had higher global QOL mean scores. A recent study in the United States showed that young adults with sarcoma had lower QOL than the general population [13]. Among functioning scales, emotional and social functioning had the lowest mean scores. Fatigue, pain and insomnia had the highest symptoms load, while dyspnoea and diarrhoea were the least common symptoms. These findings are comparable to a previous study [3]. One of the key findings of our study suggests that sarcoma survivors had an average QOL. Mean scores of the participants were relatively low on emotional, physical, social-cognitive and role-functional scales. The findings are in tune with available research evidence which reported relatively better global, physical, role and cognitive HRQoL and fewer symptoms of fatigue, pain and insomnia [14].

Our study revealed that more than one-third of survivors had depression, more than one-quarter had anxiety and slightly more than one-seventh had stress, which was consistent with previous studies [15, 16]. A recent study in Australia demonstrated that about one-fifth of the cancer survivors had depression and about half of them had anxiety, which was in contrast with the present study [17]. On the other hand, a study reported that the majority of the sarcoma patients exhibited normal or mild levels of anxiety and depression [18].

In the present study, it was found that a small number of sarcoma survivors had cognitive impairment (1% as per EORTC-QLQ and 5% as per MMSE). Conversely, previous study reported that demonstrated poorer cognitive impairment, mathematics and long-term memory among sarcoma survivors [19].

Multiple linear regression analysis found that urban residence and educational qualification of less than primary had a significant association with depression. Moreover, urban residence was found to be significantly associated with anxiety. Additionally, a family history of cancer emerged as a significant factor associated with anxiety and stress among survivors.

Treatment for sarcoma is long and chances of relapse are also there. Therefore, the survivors are in need of speaking to an expert, who can clarify their doubts on a daily basis. Running a nurse-led sarcoma clinic is a newer concept in India. The findings of the present study indicated that the survivors were satisfied with the information provided to them by the nurse and the follow-up made by the nurse for the survivors' problems. It helped the survivors in relieving their anxiety. Interaction with the nurse was important as it allowed the survivors to vent their feelings and issues in the clinic. Survivors wanted to continue this kind of interaction with the nurse in the future. Previous research evidence from the Western world showed that a nurse-led survivorship service for lymphoma patients improved documentation quality, reduced waiting times and increased patient satisfaction, demonstrating nurses' ability to deliver effective survivorship care [20].

Notably, we found that depression was not significantly associated with global QOL, which was inconsistent with various studies that demonstrated depression among breast cancer survivors was significantly associated with QOL [21, 22]. In addition, we found that urban residence was a risk factor for depression and anxiety in this sample. Educational qualification of less than primary was a significant factor predicting anxiety. And family history of cancer predicted anxiety as well as stress among sarcoma survivors. In contrast to the present study, previous research evidence demonstrated that age, gender, presenting status and time since the termination of treatments were significant determinants of anxiety and depression in sarcoma patients [18].

On analysing the concerns of sarcoma survivors using thematic analysis, the main themes identified were body image, physical appearance, low self-esteem, and acceptance; discrimination by society and support from family, relatives, and colleagues; socio-economic impact of sarcoma; marriage concern, denial of marriage and fertility issues. These qualitative insights complement the quantitative findings, providing a comprehensive understanding of the challenges faced by sarcoma survivors in India. Comparable to our findings, various studies showed that sarcoma survivors experienced several body image issues, mobility difficulties, and social isolation practices as well as they are at increased risk for reproductive difficulties, and intellectual and psychosocial issues which could potentially harm their mental health and functional QOL [5, 23, 24].

The strength of the study lies in the adequacy of a statistically calculated sample size, sufficient representation of various types of sarcoma survivors, and use of standardised tools. Focused group discussion further complemented in assessing concerns of sarcoma survivors by triangulating the data.

### Limitations of the study

The present study has limitations in terms of the convenient sample selection, and single-centric study, which limits the generalizability of the findings. It is essential to replicate this study in multiple settings. Despite the limitations, the study has unfolded the psychological issues, concerns of sarcoma survivors in India, and their satisfaction with survivorship care. The findings of the present study pave the way for redefining the role of nurses in survivorship care, enabling them to contribute significantly to the advancement of survivorship care in India.

### Recommendations for practice

There exists a need to develop and implement a comprehensive individualised survivorship care plan led by nurses for individual sarcoma survivors in developing countries like India to address the physical, emotional, and social needs of sarcoma survivors and include strategies for managing depression, anxiety, and stress, as well as addressing body image issues and social support. By recognising and addressing these issues and challenges, healthcare providers can enhance sarcoma survivor’s HRQoL and improve their overall well-being. Our study also necessitates a holistic approach to the care of sarcoma survivors that includes psycho-social support and improved communication between healthcare providers and survivors. Further research and interventions are essential to better support sarcoma survivors in their journey towards recovery.

## Conclusion

A substantial number of sarcoma survivors had an average HRQoL and experienced depression, anxiety and stress. The psychological issues of the sarcoma survivors were associated with various factors, including urban residence, education less than primary, and family history of cancer. The qualitative findings shed light on the personal experiences and struggles of sarcoma survivors, including emotional distress, physical limitations, body image concerns, societal discrimination, and concerns about marriage and fertility. Satisfaction of the survivors with individualised nursing care played a crucial role in the well-being of sarcoma survivors.

## Conflicts of interest

The authors have no relevant financial or non-financial interests to disclose.

## Funding

This research did not receive any specific grant from funding agencies in the public, commercial, or not-for-profit sectors.

## Institutional review

The study was conducted after getting approval from the Institutional Ethics Committee of All India Institute of Medical Sciences, New Delhi vide IECPG-256/24.06.2021.

## Figures and Tables

**Table 1.** Demographic and clinical profile of sarcoma survivors (*N* = 100).

Variables		Frequency
Age Mean + SD (Range)		28.76 + 12.11 (15–60)
Age group	15–30 31–45 46–60	60 28 12
Gender	Female	34
	Male	66
Residence	Rural	48
	Urban	52
Education	Post-graduation	7
	Graduation	28
	Diploma	6
	High school	31
	Middle school	22
	Less than primary	7
Marital status	Married	44
	Unmarried	56
Religion	Hindu	87
	Muslim	12
	Sikh	1
Occupation	Government job	9
	Private job	9
	Self-employed	15
	Unemployed	27
	Student	40
Type of family	Joint	64
	Nuclear	36
Monthly family income (in Rs.)	<10,001	41
	10,002–29,972	31
	29,973–49,961	14
	49,962–74,755	8
	>74,755–99,930	6
Family history of cancer	Yes	5
Diagnosis	Osteosarcoma	35
	Ewing’s sarcoma	30
	Fibrosarcoma	19
	Leiomyosarcoma/Liposarcoma	6
	Other	25
Underwent surgery		96
Received chemotherapy		87
Received radiation therapy		45
History of relapse		9

**Table 2.** HRQoL among sarcoma survivors (*N* = 100).

EORTC QoL sub-scales	Min.	Max.	Mean ± SD
Global health status/QoL	25	100	79.48 ± 16.26
Functioning sub-scales			
Physical functioning	20	100	85.53 ± 15.91
Role functioning	16.66	100	79.96 ± 20.92
Emotional functioning	17	100	77.92 ± 20.63
Cognitive functioning	17	100	88.41 ± 16.97
Social functioning	0	100	78.98 ± 21.45
Symptoms sub-scales			
Dyspnoea	0	33.33	4.66 ± 11.62
Pain	0	83.33	24.32 ± 21.24
Insomnia	0	100	13.31 ± 23.23
Fatigue	0	77.66	27.13 ± 23.64
Anorexia	0	100	13.33 ± 24.61
Nausea/Vomiting	0	83.33	11.33 ± 19.80
Constipation	0	100	11.66 ± 23.38
Diarrhoea	0	66.66	8.99 ± 18.25
Financial difficulty	0	100	22.64 ± 27.57

EORTC QoL = European Organisation for Research and Treatment of Cancer Quality of Life

**Table 3.** Satisfaction related to nursing care among sarcoma survivors (*N* = 100).

Items	Strongly agree	Agree	Neither agree nor disagree
The information provided by the nurse was important to me.	37	52	11
I was allowed to express/vent my all feelings.	25	67	8
The information provided by the nurse helped me to relieve my anxiety.	18	77	5
The explanation given by the nurse appeared correct to me.	17	64	19
The explanation/information given by the nurse was satisfactory.	11	66	23
Would you recommend the information provided to you, to someone with your kind of disease?	35	58	7
The time spent with me by nurse telephonically/offline.	7	51	42
Referral made by a nurse to an expert for my concerns.	8	67	25
Follow-up made by the nurse for my identifiable problems.	6	70	24
Would you recommend the continuation of this sarcoma clinic in the future?	41	57	2

**Table 4.** Depression, anxiety, stress and HRQoL among sarcoma survivors.

EORTC QoL sub-scales	Mean rank								
	With depression	Without depression	*p*-value^a^	With anxiety	Without anxiety	*p*-value^a^	With stress	Without stress	*p*-value^a^
Financial difficulty	57.72	49.13	0.232	63.58	47.23	0.013*	72.93	48.81	0.020*
Global health status/QoL	44.91	51.57	0.390	43.40	52.28	0.211	55.50	50.12	0.629
Functioning sub-scales									
Physical functioning	53.78	49.88	0.614	43.03	52.37	0.189	47.36	50.74	0.762
Role functioning	51.03	50.40	0.933	47.93	51.14	0.640	57.30	49.97	0.486
Emotional functioning	28.56	54.68	0.001*	30.23	55.57	<0.001*	22.71	52.59	0.008*
Cognitive functioning	38.72	52.17	0.056	31.85	54.59	<0.001*	23.00	52.05	0.004*
Social functioning	44.69	51.61	0.361	41.68	52.71	0.112	36.29	51.57	0.160
Symptoms sub-scales									
Dyspnoea	43.50	51.83	0.080	48.50	51.00	0.566	50.64	50.49	0.982
Pain	55.66	49.52	0.423	64.78	46.93	0.011*	71.07	48.95	0.044*
Insomnia	59.47	48.31	0.079	68.63	45.58	<0.001*	72.42	48.55	0.013*
Fatigue	62.50	48.21	0.067	75.53	44.24	<0.001*	82.29	48.11	0.002*
Anorexia	52.56	50.11	0.693	61.98	47.63	0.012*	64.93	49.41	0.083
Nausea/Vomiting	50.47	50.51	0.995	68.10	46.10	<0.001*	78.29	48.41	0.001*
Constipation	49.41	50.71	0.823	58.15	48.59	0.073	54.86	50.17	0.575
Diarrhoea	46.13	51.33	0.362	51.93	50.14	0.734	47.86	50.70	0.729

a *U* = Mann–Whitney *U* test; *p* = significance level

EORTC QoL= European Organisation for Research and Treatment of Cancer Quality of Life

* Statistically significant where *p* < 0.05 and two tailed

**Table 5.** Multiple linear regression analysis of the factors influencing depression, anxiety and stress among sarcoma survivors.

	*B*	Standard error	*β*	*t*	*p*
Depression					
Constant	1.225	1.003	–	1.220	0.225
Age	0.001	0.010	0.011	0.076	0.940
Gender	0.283	0.193	0.153	0.466	0.146
Residence	0.398	0.187	0.227	2.126	0.036*
Educational qualification	−0.128	0.063	−0.228	−2.040	0.044*
Occupation	0.043	0.092	0.064	0.470	0.640
Type of family	0.163	0.177	0.089	0.922	0.359
Diagnosis	−0.055	0.038	−0.158	−1.450	0.151
Family history of cancer	−0.687	0.398	−0.171	−1.724	0.088
Anxiety					
Constant	3.478	1.420	–	2.449	0.016*
Age	−0.017	0.015	−0.158	−1.148	0.254
Gender	−0.037	0.273	−0.014	−0.135	0.893
Residence	0.667	0.265	0.261	2.518	0.014*
Educational qualification	−0.032	0.089	−0.039	−0.358	0.721
Occupation	0.144	0.130	0.146	1.111	0.270
Type of family	0.041	0.250	0.015	0.164	0.870
Diagnosis	−0.101	0.054	−0.198	−1.870	0.065
Family history of cancer	−1.623	0.564	−0.277	−2.879	0.005*
Stress					
Constant	1.643	0.761	–	2.158	0.034*
Age	−0.004	0.008	−0.078	−0.549	0.585
Gender	−0.033	0.146	−0.023	−0.222	0.825
Residence	0.183	0.142	0.138	1.292	0.200
Educational qualification	0.009	0.048	0.021	0.185	0.853
Occupation	0.030	0.070	0.059	0.436	0.664
Type of family	0.182	0.134	0.132	1.360	0.177
Diagnosis	−0.038	0.029	−0.143	−1.311	0.193
Family history of cancer	−0.890	0.320	−0.290	−2.942	0.004*

* Statistically significant where *p* < 0.05 and two tailed; B = Unstandardised coefficient; *β* = Standardised coefficient

**Supplementary Table 1.** Trigger questions and themes to explore the concerns of sarcoma survivors and their satisfaction with survivorship care.

Sl. No.	Trigger questions	Themes
1	Please tell how you feel about the changes in your body/physical appearance after the surgery	Body image, physical appearance, low self-esteem and acceptance
2	How do you feel about the way your family, relatives and society after your cancer was diagnosed?	Discrimination by society and support from family, relatives and colleagues
3	How has cancer affected your job/employment ability, has it caused any difficulty?	Socio-economic impact of sarcoma
4	What is your view regarding the marriage, pregnancy and future planning please explain.	Marriage concerns, denial of marriage and fertility issues
5	How has been your experience during the treatment at AIIMS?	Quality of services, amenities and waiting for an hour
6	What improvement would you like to see in the behaviour of doctors, nurses, health care professionals of AIIMS hospital for your better care?	Satisfaction with the doctor, nurses and healthcare professionals
